# Electronic Tongue—A Tool for All Tastes?

**DOI:** 10.3390/bios8010003

**Published:** 2017-12-31

**Authors:** Marta Podrażka, Ewa Bączyńska, Magdalena Kundys, Paulina S. Jeleń, Emilia Witkowska Nery

**Affiliations:** 1 Institute of Physical Chemistry, Polish Academy of Sciences, Kasprzaka 44/52, 01-224 Warsaw, Poland; mpodrazka@ichf.edu.pl (M.P.); e.baczynska@nencki.gov.pl (E.B.); mkundys@ichf.edu.pl (M.K.); pjelen@ichf.edu.pl (P.S.J.); 2 Laboratory of Cell Biophysics, The Nencki Institute PAS, Pasteur Street 3, 02-093 Warsaw, Poland

**Keywords:** electronic tongue, sensor arrays, pattern recognition, chemometrics

## Abstract

Electronic tongue systems are traditionally used to analyse: food products, water samples and taste masking technologies for pharmaceuticals. In principle, their applications are almost limitless, as they are able to almost completely reduce the impact of interferents and can be applied to distinguish samples of extreme complexity as for example broths from different stages of fermentation. Nevertheless, their applications outside the three principal sample types are, in comparison, rather scarce. In this review, we would like to take a closer look on what are real capabilities of electronic tongue systems, what can be achieved using mixed sensor arrays and by introduction of biosensors or molecularly imprinted polymers in the matrix. We will discuss future directions both in the sense of applications as well as system development in the ever-growing trend of low cost analysis.

## 1. Introduction

The widely accepted definition of electronic tongue systems states:

“The electronic tongue is an analytical instrument comprising an array of nonspecific, low-selective, chemical sensors with high stability and cross-sensitivity to different species in solution and an appropriate method of PARC and/or multivariate calibration for data processing” [1].

**Figure 1:**
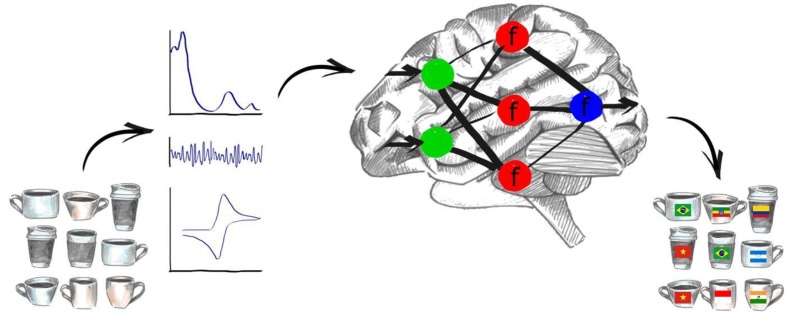
Working principle of an electronic tongue system: Physical, chemical and biochemical properties of the samples are measured by means of an array of sensors, which translate those specific attributes to an analytical signal (optical, electrophysiological, electrochemical etc.). So obtained data are then analysed by means of chemometric techniques or neural networks, which provide final information about the sample—for example discriminate coffee samples by their geographical origin.

Their working principle, presented schematically on Figure 1 was inspired by biological recognition in which information is gathered with the use of arrays of non-specific sensors in the nose or tongue and the data is subsequently processed in the brain. Electronic tongues use chemometric methods and artificial intelligence to achieve a similar goal, i.e., discriminate, identify or quantify the sample [2,3].

First sensor arrays of this kind appeared in the 1990’s and were mainly applied to the analysis of ions and heavy metals [4,5] as well as evaluation of taste [6] and spoilage [7] of food products. 

Mimicking human taste is advantageous in situations when human expert panels cannot or should not be applied, because of:

- process conditions—as in case of automatic process control especially on an industrial scale;
- poisonous/extreme condition samples—e.g., repetitive tasting of drugs and pharmaceuticals;
- economic reasons, defined in terms of time or financial expenses;

Even though publications regarding medical [8] and industrial [9] uses of such systems surfaced not long after the first appearance of the electronic tongue itself, until today the better part of applications relates to the literal meaning of the word ‘tongue’. In this review, we would like to show that mixed sensor arrays have in fact much broader use and by any means should not be associated only with food, beverages and taste of pharmaceuticals. For that, we need to think of an electronic tongue not as a physical object containing several integrated sensors but rather as a set of sources of data obtained from a liquid sample, regardless to their nature (spectroscopic, electrochemical etc.). 

Already upon appearance of electronic tongues, scientists drew attention to the advantages of multivariate analysis and its meaning to analytical chemistry. It was shown that the performance of individual sensors can be greatly improved in terms of the limit of detection and selectivity by the simple inclusion of data from seemingly not related sensors [10]. In the end, lion’s share of sensors is not selective and provides hidden information regarding other components of the sample [11] the decision whether we should benefit from it or not is up to us. 

In the upcoming sections, we will first take a closer look at different types of sensors employed in electronic tongue systems and the data they produce. Later let us ponder on the possibilities brought by, recently popular, unconventional methods of fabrication of low-cost sensors. We will also consider what can be gained by the inclusion of biosensors and sensors based on molecularly imprinted polymers. To sum it up, in the end, we would like to present the main applications, both from alimentary and pharmaceutical sectors and outside of it. 

## 2. Sensors Types and the Resulting Data

Sensors employed in the electronic tongue systems range from electrochemical (potentiometric, amperometric, voltammetric, impedimetric, conductometric) through gravimetric to optical (absorbance, luminescence, reflectance etc.) [3,12].

An ideal matrix should be composed of both selective and cross-sensitive sensors. According to IUPAC report, cross-sensitivity is an ability of a sensor to respond reproducibly to a number of different analytes in the solution [1]. This trait is observed in biological recognition systems which are composed of partially or non-selective sensors of little variety (few dozens in case of taste, to around hundred for smell) [1]. Complex data patterns provided by gustatory and olfactory receptors are comprised of multiple, individual cross-sensitive responses [13]. It is truly astounding that based on all that data, which in laboratory conditions would be considered as noise, biological systems are able to recognize thousands of compounds, sometimes very similar in structure (e.g., stereoisomers) and with detection limits in the range of 1 to several hundred ppb [14,15].

In terms of an electronic tongue, Ciosek et al. [16] already proved that inclusion of cross-sensitive sensors into the array of ion-selective electrodes allows higher accuracy of discrimination. On the other hand, based on our own experience [17], the artificial matrix should not be composed of only cross-sensitive sensors, as this kind of electronic tongue has lower capabilities of prediction than a mixed selective/cross-selective matrix. 

Dynamic components of the measured signal should not be underestimated as well. Electronic tongues operated in automated flow systems can provide additional kinetic information about the sample. As the speed of sensors response versus primary and secondary species can vary, dynamic measurements can have a positive impact on both modelling and reduction of the influence of interferents [3,13]. 

Usually, electronic tongue systems are built from few to dozens of sensors of a single type, the most common being potentiometric and voltammetric [18]. Even though both are electrochemical, voltammetry on the contrary to potentiometry involves the flow of a current between the electrodes and thus in most cases results in more complex data. Even though voltammetry can provide higher order information also regarding the kinetics of the reaction, it is only applicable to redox-active species. On the other hand, potentiometric sensors are sensitive to charged molecules. Given those complementary characteristics, it is not surprising that hybrid electronic tongues combining both techniques were already proposed, publications include systems for classification of fermented milk [19], analysis of beer fermentation [20], determination of levels of NaCl, NaNO_3_ and KNO_3_ in minced meat [21] and detection of trinitrotoluene TNT [22].

Fusion of data from different sensor types can help to determine the fingerprint of a given substance allowing better classification and identification even if the background of the sample changes drastically. It can be achieved thanks to multitransduction, that is by measuring of different properties of the same sensor, as in case of quantification of electrochemical and optical changes observed for porphyrin electropolymer films [23]. Other possibility is simple merging of data from physically separated optical and electrochemical sensors [24,25]. In the trend of replacing human sensory panels electronic tongue systems are often combined with sensors for gaseous samples, so called electronic noses [26,27,28,29], in this way better mimicking traditional way of analysis (evaluation of smell, taste and colour of food samples). 

Even though nowadays running out of computing power does not seem as a serious treat even in case of sensor arrays composed of few dozens of sensors, excessive increase of the number of transducers can still involve problems. We should remember that integration of multiple sensors will result in redundant information. It can be useful: improve precision, stability and provide better separation of clusters [30] or just the opposite: increase the number of misclassifications and decrease the distances between groups. We have shown, that better classification could be obtained by the direct plotting of sensor response from 3 out of 4 sensors composing the matrix, than by applying Principal Component Analysis (PCA) to all the data [31]. This is a classic example how important is a careful analysis of the Loadings Plot in case of PCA. It also shows the significance of iterations during the data analysis process. 

Too numerous matrixes together with neglectful data treatment can easily result in over-fitting of the developed models, which instead of recognizing trends will start to find the perfect match for the training data set. Fortunately, more and more editors request inclusion of the original data in the digital versions of published manuscripts. We can all hope that such actions will result in better models, which will be jointly developed by cooperative work of the scientific community. It will also allow to perform data fusion not only with different types of sensors as it happened until today but also by combination of the data obtained in different research groups around the world. 

Last but not least let us quote a sentence which should always be remembered when dealing with populous data: “If you torture your data long enough, they will tell you whatever you want to hear” [32].

## 3. Commercial Systems

**Figure 2:**
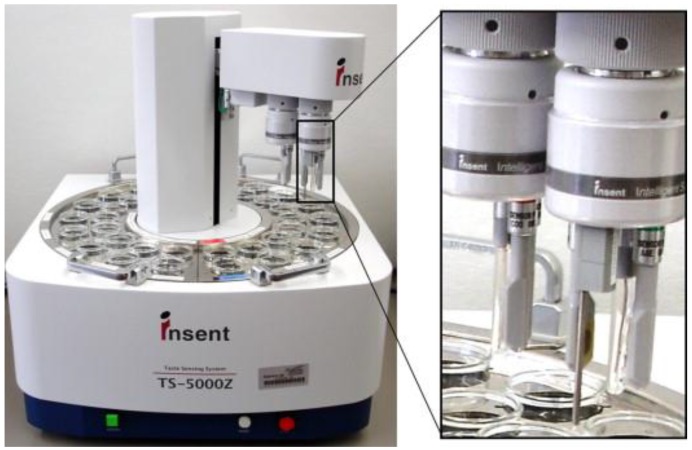
TS-5000Z taste sensing system, Intelligent Sensor Technology Inc., Japan. Reprinted with permission from [43] ^©^Elsevier).

Given the numerous advantages and a wide range of applications of electronic tongue systems, their commercialization was only a matter of time. The first electronic tongue that became available on the market was constructed by Toko and co-workers [6]. SA402B and TS-5000Z (Figure 2) Taste Sensing Systems (Intelligent Sensor Technology Inc., Atsugi-shi, Kanagawa, Japan) are based on Toko’s idea and consist of 7 potentiometric electrodes with lipid-polymeric membranes [33]. The latter is the newest model, designed for both use in a laboratory and as a part of a network system in quality control. It enables detection of five basic tastes and also flavour attributes like “sharpness” and “richness” [34]. Both systems are mainly used to determine and quantify the intensity of each taste in food products [35,36,37] and pharmaceuticals [38,39,40,41]. A review concerning the application of commercial systems for evaluation of taste masking properties was recently published [42]. 

Another widely distributed commercial tongue is Astree II (Alpha MOS, Toulouse, France). It is composed of 7 ion-selective field effect transistors (ISFET) and is dedicated to discriminate samples according to their taste properties. Literature is rich in examples of its application in quality control [43,44], food recognition [45,46,47], taste assessment [48,49], process monitoring [50,51] and pharmaceutical industry [41,52]. Apart from taste system Alpha MOS company offers also electronic nose and electronic visual analyser for colour and shape analysis [53]. 

Two other electronic tongues are available on the market. The Multiarray Chemical Sensor (McScience Inc., Suwon, Korea) build of PVC and polyurethane membranes selective towards H^+^, Na^+^, K^+^, Ca^2+^, NH_4_^+^, NO_3_^−^, Cl^−^ [3] and Sensor System (St. Petersburg, Russia) comprised of 7 potentiometric ion-selective sensors [54]. In contrast to previously presented sensors, the literature concerning the application of those systems is rather scarce.

Besides commercial systems, there is also the possibility to order taste assessment service. Aissy Inc. from Tokyo, Japan provides an accurate analysis of food products using its original taste sensor and compatible software which can be helpful for the development of new products in the food industry, especially in case of researchers less familiar with sensor matrix technology [55]. 

## 4. Nature Inspired Sensors

Biosensors and chemical sensors based on advanced recognition methods, so-called molecularly imprinted polymers (MIP) can be applied in the sensor matrix using the same transduction properties as in case of traditional chemosensors. Even though biosensors are often given as an example of extreme specificity, many undergo reaction with a group of substances (sugars, phenols etc.), they can often also provide information about a vast number of other species, namely inhibitors. Although MIP sensors are designed to recognize a specific molecule, they frequently also provide cross-selectivity and thus are perfectly suited for electronic tongue applications. 

### 4.1. BioElectronic Tongue

This new approach has been presented in 1999 when the array was equipped with several biosensors [56]. High selectivity and specificity can provide added value for the given electronic tongue, in which other sensors will help to diminish the impact of a complex and changing background or interferents. Contemporary biosensors are created on the base of a vast range of recognition elements including nucleic acids and aptamers, antibodies, cells, phages as well as enzymes, the latter being the most popular until today. 

The enzymatic modification allows acceleration of the directed recognition reaction of the sensor. Moreover, this kind of sensors enables collection of additional kinetic information. Both acceleration of the catalysed reaction and inhibition of the enzyme give valuable background information and taken into consideration during chemometric analysis can significantly improve the final discrimination. Due to desirable characteristics, biosensors found application in electronic tongue systems used in science, food-industry and agriculture. Both tyrosinase and glucose oxidase were successfully incorporated into lipid membranes which helped to preserve their activity and improved sensitivity of the overall matrix [57]. The same research group used a similar method of entrapment to fabricate sensors based on both chemical catalyst (bisphthalocyanine) and enzymes (tyrosinase and laccase). This three-electrode matrix was successfully applied for quantification of 6 different phenolic compounds, with detection limits of 10^−7^–10^−8^ mol/L as well as discrimination of wine musts based on their Total Polyphenolic Index [58]. Authors point out that the nature of the enzyme which conditions its incorporation in the Langmuir–Blodgett films as well as enzymatic specificity and kinetics of the reaction were crucial for the successful functioning of the electronic tongue. 

An eye-catching application of a bioelectronic tongue for the quality assessment of chemical plant’s wastewater was described recently by Czolkos et al. [59]. The matrix was composed of 8 sensors, 6 of them being based on enzymes. The proposed bioelectronic tongue was able to predict values of one of two evaluated inhibition tests (based on bacteria *Vibrio fischeri*) and all three global organic pollution parameters (total organic carbon, chemical and biochemical oxygen demand). The analysis was performed using a flow-injection system and authors took time to analyse the impact of dynamic components of the signal (taking into consideration entire signal as well as separate parameters such as peak area and height plus slope). In this case, flow conditions will not only influence the electrochemical response by direct changes in the diffusion profile but also as a consequence affect the interaction between the enzyme and the substrate and/or inhibitor. Data from steady-state measurements were best suited to predict total organic carbon and biochemical oxygen demand, whereas in case of other parameters dynamic conditions were more adequate.

Bioinspired electronic tongues were also applied for the analysis of pesticides, which are traditionally assessed by means of enzyme inhibition assays (usually based on acetylcholinesterase). The biggest problem of the inhibition assay is the complete lack of specificity as the enzyme can be inhibited by heavy metals, a wide range of pesticides and other species. In this case electronic tongue systems can inform not only about the presence of a harmful substance but also help to predict its type. This kind of systems was already applied to both steady-state [60] and automated, in-flow analysis [61] of real samples.

More information regarding bioelectronic tongues can be found in [62].

### 4.2. Molecular Imprinted Polymers

Enzymatic biosensors can be highly sensitive and provide both selective and cross-selective information to a range of substrates and inhibitors but often suffer from slow deactivation. Apart from chemical catalysts, Molecularly Imprinted Polymers (MIP) were proposed as substitutes. In this case, monomers are co-polymerized in the presence of the target molecule. After the removal of the imprinted molecules, a final polymer is able to selectively rebind them in the so formed cavities [63]. 

Forming an array of MIP sensors takes advantage of their high cross-reactivity and usually limited selectivity [64]. As a variety of MIP sensors can be prepared in the same manner, a largely diversified matrix can be fabricated with relative ease and at low expense. Applications of this kind of multielectrode systems were reviewed recently in [63,64]. Although the validity of application of molecularly imprinted polymers for protein recognition is a subject of scientific debate, reports indicate successful discrimination of four different of proteins [65]. Changes in the tertiary and quaternary structure of proteins can be easily induced by even small variations in the environment thus completely preventing molecular recognition using imprinted cavities. Fortunately, this effect can be somewhat diminished by application of a sensor matrix. 

### 4.3. Brain-Machine Interfaces and Animal Models

The concept of in vivo/ex vivo electronic tongues takes bioinspired approaches a step further, as in this case matrix can be formed of isolated taste receptors, gustatory cells or even the whole animal, by connection of electrodes to the specific part of the brain. In case of the brain-machine interface, the scientific challenge is to decode the information resulting from the cerebral analysis of the signal sent from the tongue of the animal. Taste analysis using those approaches is described in detail in [66] Chapters 10–15.

Even though brain-machine interfaces are investigated for innovative artificial prostheses that can be controlled directly by the brain they can be also used for fundamental studies on taste transduction mechanisms and cell-to-cell communication. In this kind of systems, the animal is considered a black box with tongue as the sensing element and appropriate brain region as the processing unit producing signals composed of spikes and local field potentials. This kind of systems were already applied to distinguish between quinine, sucrose, NaCl and HCl corresponding to basic flavours [67,68]. 

In a more ethical approach, traditional behavioural studies can be used to develop a model for assessment of taste masking of pharmaceuticals [69], more examples of which are reviewed in [42]. 

## 5. Low-Cost Sensor Arrays

Electronic tongue systems are often built to provide general information about the sample and detect abnormalities, being it a spoiled food product, polluted water sample or an alcoholic beverage aged with the aid of caramel. In many cases functioning of such systems is not ideal, or they wider application is difficult because of strict regulations concerning for example sensors intended for commercial clinical use. Nevertheless, even in such circumstances, electronic tongue systems could serve as a perfect screening device, providing preliminary information regarding the need of more specialized analysis or indicating the source of the problem. For this kind of analysis, low-cost sensor arrays would have the biggest chance to achieve commercial success.

For a bit more than a decade sensor platforms are undergoing a true revolution as in search of a more available, less expensive and often disposable alternatives to sophisticated equipment sensors are being transferred to substrates such as paper, plastic or even thread [70,71,72,73]. Together with open source detection systems, which can be built from scratch using off-the-shelf components [74] or thanks to the use of existing equipment such as smartphones [75,76] this kind of systems can easily spread and thus possess a great potential for commercialization. 

**Figure 3:**
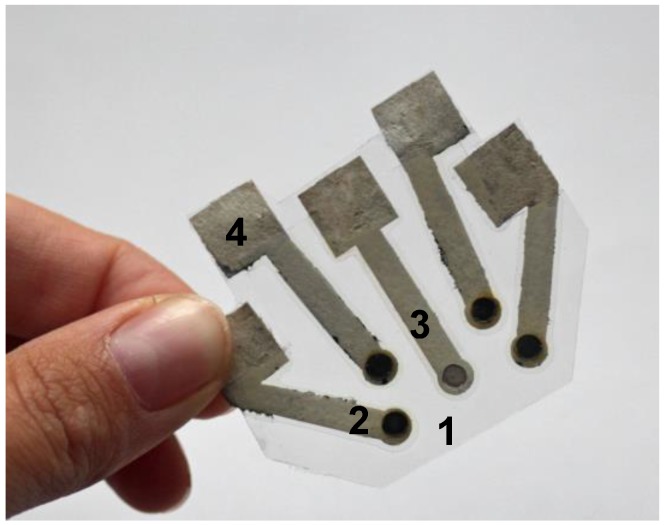
Paper-based electronic tongue system applied for discrimination of beer and wine samples 1. lamination sheet serving as support, 2. ion-selective electrode, 3. reference electrode, 4. electrical connections (reprinted with permission from [17] ^©^Elsevier).

The fact that electronic tongue systems have great diversity of applications creates a significant demand for their further development especially in the trend of low cost diagnostics. Recently we presented the first electrochemical electronic tongue with integrated reference electrode based on paper [31]. The final system (Figure 3) included four working silver electrodes with a paper based Ag/AgCl reference all integrated in one miniaturized device and was successfully applied for the analysis of beer and wine samples [17]. Paper based colorimetric tests were also joined to form an array able to identify eleven common organic solvents [77]. Each of the sensors would change colour between blue to red upon contact with the solvent and the detection could be performed by means of a scanner.

Another low cost electronic tongue system was introduced by Garcia Breijo et al. [78]. In this case different electrode configurations together with voltammetry and impedance spectroscopy were tested in order to quantify trinitrotoluene TNT. 

Microfluidic devices are also an interesting direction of development of sensor arrays, as they can work with low volumes of liquid, can be self-contained and thanks to technology development are already being fabricated in mass, which greatly diminishes their cost [79]. A microfluidic electronic tongue capable of distinguishing of basic flavours was recently proposed [80].

Low cost sensors can perform analysis on micro- and macro scale and be used individually or in arrays forming complex but inexpensive electronic tongue systems. A list of already developed sensors which could be integrated in such a way is almost endless but to name just a few we can think of paper based systems both colorimetric [81] and electrochemical [82], electrodes fabricated from CDs [83] or parts of integrated circuit chips [84]. An interesting sensor architecture, with great potential of application is the tattoo-based sensor, which was recently introduced as means of monitoring of epidermal pH, NH_4_^+^ and Na^+^ levels. These systems, were already applied to different areas of the human skin (i.e., hand, back, foot, neck) for real-time monitoring of aforementioned ions during perspiration [85,86,87] and in principle could be applied in series taking advantage of electronic tongue technology.

**Figure 4:**
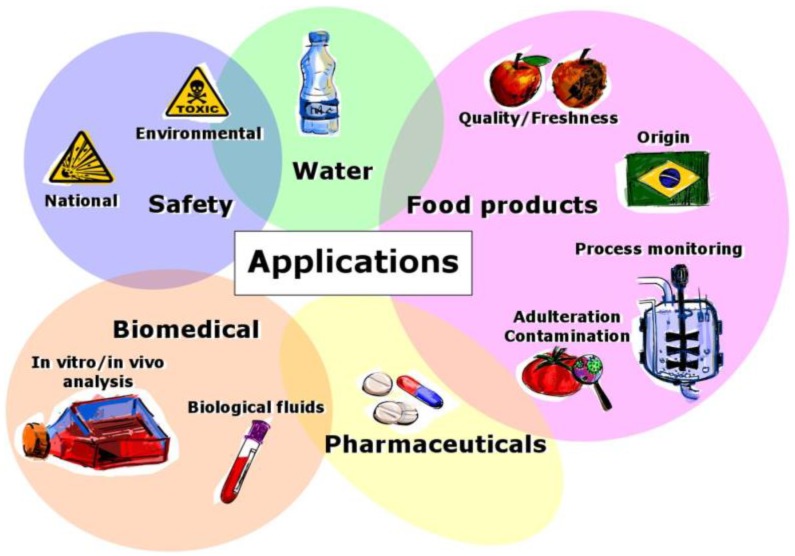
Wide range of applications of electronic tongue systems.

Subsequent section will describe the most relevant applications of electronic tongue systems, which are graphically resumed in Figure 4.

## 6. Main Applications

Distinguishing between different tastes is the basic task of the human tongue. That is why the most common applications of its artificial analogue are the flavour assessment and detection of compounds responsible for the taste perception. It may seem like a trivial task but due to a synergistic/suppression effect, the actual taste of a food product may not be possible to assess even if all individual components were quantified. 

The first electronic tongue was designed to recognize five basic tastes: sweet, sour, salty, bitter and umami [88]. Since then, sensors have been developed to analyse and classify flavours of multicomponent mixtures [89] becoming a versatile tool widely used in food analysis, environmental monitoring and pharmaceutical industry.

### 6.1. Foodstuffs

Nowadays, food products are controlled during the entire food supply chain to ensure their quality and comply with the safety requirements. A wide range of analytical methods is used in the food industry (e.g., liquid chromatography, IR or UV spectroscopy) but, despite all the advantages they are time-consuming, require expensive equipment and skilled personnel. As those characteristics are incompatible with stages of the modern food supply chain (production, transport, distribution) and validation by the consumer, new screening methods are sought after. 

The electronic tongue appears as an ideal device for that purpose as it enables fast, precise and direct analysis [90]. Moreover, it can be applied for the automatic on-line monitoring during food processing. This section describes the most relevant applications of taste sensors, such as sample recognition/origin tracing, process monitoring and quality control.

#### 6.1.1. Recognition and Origin Tracing

Food authentication is one of the main concerns of the food industry. All the products need to comply with labelling in terms of brand, origin, ingredients and production process. Information about the geographical origin is the most crucial as it one of the major factors affecting the price. Authenticity testing using electronic tongues was recently reviewed in [91]. 

Alcohols are one of the most frequently studied products in terms of the brand and origin recognition. Blanco and co-workers presented a portable electronic tongue system based on disposable screen-printed electrodes capable of distinguishing between different types of Lager beer, predicting its colour and alcoholic strength(accuracy of 76% and 86% respectively) [92]. 

A hybrid electrochemical-optical system was applied for the discrimination of 25 genotypes of white wine grapes and their differentiation from 3 reference grape juice samples. A set of seven ISFET sensors (ion-selective field effect transistor) provides information about the presence and concentration of some key ions, redox species and organic compounds in the grape juice while the absorbance spectra enable colour estimation [93].

Recently, we [17] described a novel paper-based potentiometric electronic tongue (Figure 2) with an integrated reference electrode which served as a tool for beer and wine analysis. Proposed sensor was able to discriminate beers from 19 brands and 12 different types. Moreover, it allowed detection of stabilizers, antioxidants, dyes and substances added during the fermentation process. In case of wine, samples could be classified according to the grape variety. A significant advantage of the system is its low cost, versatility and ability to work with microliter sample volume. 

Other groups of products frequently studied in terms of label authentication are oils, dairy products, non-alcoholic beverages, honeys, teas and coffees. A potentiometric electronic tongue containing cross-sensitive lipid membranes was applied to discriminate monovarietal extra virgin olive oils according to olive cultivar and geographic origins. Recorded signals were analysed by meta-heuristic simulated annealing algorithm together with the LDA resulting in sensitivity above 97% [94]. Escriche et al. fabricated a system made of metals (Au, Ag and Cu) and metallic compounds (Ag_2_O, AgCl, Ag_2_CO_3_, Cu_2_O) able to classify honey by its botanical origins. A notable correlation of the response with physicochemical parameters confirmed the effectiveness and reliably of the described method [95]. 

Garcon and co-workers developed a new taste system by coupling surface plasmon resonance imaging (SPRi) with cross-reactive sensor arrays. It is worth mentioning that 11 sensing receptors were prepared by mixing only two disaccharides. This, seemingly simple sensor matrix successfully discriminated different milk samples (UHT pasteurized cow milk, unpasteurized cow milk, soy milk, soy milk with chocolate and rice milk) and SPR provided additional information about the adsorption and desorption kinetics of the studied compounds. Continuous evaluation of the data allowed automatic exclusion of abnormal signals from defective sensors [96].

#### 6.1.2. Evaluation of Food Quality and Freshness 

Quality of food products is of ever-growing interest of consumers. Even though regulations concerning the safety of food exist, there are still cases of fraud dictated by the desire of commercial profits. 

The quality assessment of wine is usually carried out by a trained sensory panel through evaluation of the aroma and taste properties. Ceto et al. demonstrated the capability of the electronic tongue to mimic the human taste perception by enabling evaluation of wine testing descriptors [97].

Total phenolic content as well as identification of specific phenolic classes are yet another often analysed factors. Phenols strongly contribute to the colour, astringency, bitterness and act as a preservative during the aging process of wine, therefore it is not surprising that several bioelectronic tongues were proposed for quantification of phenolic compounds in this type of beverage [58,98]. Similar aging process contributes to attributes of beer which was evaluated in terms of flavonoid content [99]. Phenols are also responsible for beneficial health effects and taste profile of olive oils. An array of polypyrrole modified screen-printed electrodes has been used for the quantification of total polyphenol content and discrimination of individual phenolic compounds in 18 extra virgin olive oil samples [100].

In case of the meat and fish products, freshness evaluation is highly important because rotten or spoiled products may negatively affect the health of consumers. Therefore, a multi-sensor system based on modified screen-printed electrodes has been tested as a possible tool for the detection of ammonia and putrescine (toxic diamine produced during decomposition of amino acids) in a powdered beef extract. Proposed sensor matrix showed an excellent sensitivity towards amine compounds (LOD of 1.85 μmol/L for ammonia and 0.34 μmol/L for putrescine) [101].

New, interesting application of a taste sensing system is the detection of gluten in different foodstuffs. Currently, a wide range of products available in the market are labelled as “gluten-free.” However, detection of gluten is mostly done by expensive and time-consuming techniques such as mass spectroscopy and polymerase chain reaction. Daikuzono et al. reported an alternative method for the determination of gliadin (one of gluten-forming proteins) based on a microfluidic impedance electronic tongue [102]. The device was capable to discriminate gluten-free products from the ones deliberately contaminated with trace amount of gluten and detect gliadin concentrations as low as 0.005 mg/kg.

A voltammetric electronic tongue was applied for quantification of physical and chemical attributes of cooked rice, such as softness, stickiness, sweetness and aroma. PCA and FFT (fast Fourier transform) methods were used to assign each attribute to a specific electrode and frequencies of the voltammogram resulting in final correlation coefficients above 0.9 [103].

Apart from the quality monitoring of the products just released on the market, control of changes appearing during the storage time is also needed. Evaluation of freshness of pork loin stored for 10 days under refrigeration was performed with a potentiometric electronic tongue. Results were compared with routine physicochemical, microbial and biochemical measurement and allowed to estimate the time elapsed in relation to meat degradation [104]. A similar technique was also used to study samples of unsealed pasteurized milk [105]. 

#### 6.1.3. Process Monitoring

**Figure 5:**
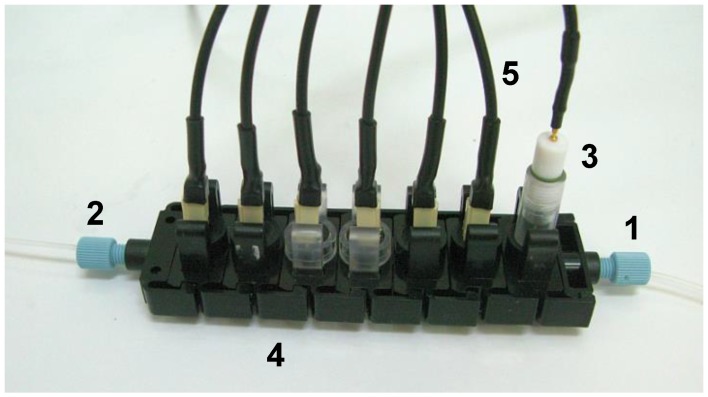
Example of an electronic tongue system with individual sensing modules applied for the analysis of fermentation: 1. inlet, 2. outlet, 3. reference electrode, 4. single modules with individual sensors, 6. electrical connections (reprinted with permission from [109] ^©^Elsevier).

Electronic tongues can be also successfully applied for the monitoring of changes occurring in the composition of foodstuff during its production. One of the most significant applications is the fermentation process. Continuous control of the fermentation helps to avoid unfavourable deviations, detect microbiological contamination and ensure the feasibility of the processes [106]. In many cases, mixed cultures of bioorganisms are used, which trigger themselves at specific time points of the process. Adding to that variations in the composition of prime matter used in the process it is difficult to name any other analytical technique, which could be reliably applied to analyse fermentation samples. Electronic tongue systems are currently the only systems able to deal with such samples, which are extremely complex, present huge variations in the background composition and have to be studied in detail as in some cases lack of a certain nutrient can stop the process or lead to unwanted by-products [107,108,109]. Example of such system applied for analysis of extremely complex samples from methane fermentation is presented in Figure 5. 

In order to simplify the analysis of carbohydrates during the optimization of the production process, an electronic tongue formed by metals oxy-hydroxide/MWCNT modified electrodes has been developed. Accurate quantification of galactose, glucose, xylose and mannose content in sugarcane bagasse is important for the usage of proper microorganisms for the production of second generation ethanol [110]. Another type of e-tongue based on miniaturized, potentiometric and voltammetric sensors was employed for the monitoring of beer fermentation. Combination of the two different electrodes arrays enabled improved classification capability of the system as compared to data analysed separately [20]. 

Another production step often following the fermentation is aging, which is known to improve the quality of alcoholic beverages such as wine. As the cost of wooden barrels highly contributes to the price alternative ways are sought after. Rudnitskaya et al. studied the effect of wine maceration with inexpensive oak chips using a potentiometric electronic tongue. The sensor matrix was capable to distinguish artificial wine solutions and Cabernet Sauvignon wine macerated with four different types of oak chips. The proposed system was able to assess phenolic content which depended on the type of oak chips added to the wine [111].

An interesting example of application of sensor arrays is the monitoring of a ham-curing process. Such samples were recently discriminated according to the curing time with 100% effectiveness by means of a potentiometric matrix [112].

#### 6.1.4. Detection of Adulteration and Contamination

Presence of undesirable substances not mentioned on the label is not only a criminal offense but may also pose a serious risk to the consumer’s health. Thus, development of fast, precise and low-cost methods for the detection of contaminations in food products is of the highest importance.

Recently, an automated hydrodynamic bioelectronic tongue based on genetically modified acetylocholinesterade was applied for quantification of pesticide (chlorpyriphos-oxon and malaoxon) mixtures in milk [113]. In another study, a voltammetric electronic tongue enabled quantification of formaldehyde, urea and melamine in milk with limits of detection (10.0, 4.16, 0.95 mmol/L respectively) below the limit of the recommended tolerable intake dose [114].

Campo et al. described a voltammetric electrode array able to predict levels of the most widely used curing agents, nitrate, nitrite and chloride in minced meat and in saline solution [115].

Apart from the detection of harmful compounds, assessment of the adulterants deliberately added to reduce cost of the production and maintain high price of the final product is a crucial issue concerning the food industry. One of the examples includes the examination of virgin olive oil adulteration, presented by Apatrei. An array of modified carbon paste electrodes was employed for the evaluation of the percentage content of edible oils (sunflower, soybean and corn oils) in olive oil samples. Data obtained after processing of voltammetric signals using PLS (Partial Least Squares) discriminant analysis and regression, demonstrated the ability of the sensor to classify precisely the aforementioned adulterant oils with a concentration level below 10% [116].

More detailed information about application of electronic tongues in food adulteration control can be found in the review articles dedicated to this subject [91,117].

Pesticides form another class of compounds of interest when it comes to food quality assessment and detection of contaminants. Potential presence of pesticides forces additional quality control in case of vegetables and fruits certificated as ecological. Many analytical methods have been developed to detect these compounds such as chromatographic and spectroscopic techniques however, all of them require professional equipment and pre-treatment of the sample. Electronic tongue systems was already successfully applied in this field, allowing monitoring of organophosphate pesticides at nanomolar levels [118].

### 6.2. Water Analysis

Analysis of water samples is another important field in which electronic tongues could possibly displace traditional analytical methods. Similarly, to foodstuff monitoring the attention of the researchers is focused on the development of reliable and low-cost tools for the authentication and quality assessment especially in case of drinking water. 

Quality evaluation is required not only throughout the production line but also in the final bottled products due to the fact that adulterations can appear at any stage of the transport or storage. Recently we [31] described a low-cost paper-based sensor able to differentiate water samples collected from the tap or lake from commercially bottled products and mineral water samples obtained directly from the spring. 100% of correct classifications and fact that the sensor was fabricated from inexpensive, readily available materials makes it a very promising tool for adulteration control of bottled water.

One of the main factors in the selection of products by consumers is the brand. Not only food but also water products from certain manufactures possess specific composition and flavour attributes. A potentiometric e-tongue with cross-sensitivity lipid membranes was utilized to distinguish between 34 water samples including still, sparkling and flavoured mineral waters. Analysis of the obtained signals with K-folds cross-validation technique resulted in the 96% mean correct classification rate. Moreover, physiochemical parameters like pH and conductivity were quantitatively estimated [119].

### 6.3. Taste Masking of Pharmaceuticals

Efficient treatment of infections and diseases is based on the usage of Active Pharmaceuticals Ingredients (API) such as paracetamol, ibuprofen, diclofenac, caffeine and many more [120]. The human sense of taste can distinguish several different flavours, inherently associated with pleasure or dislike. Unfortunately, API taste is usually associated with bitterness and thus considered unpleasant. A popular approach is based on coating tablets and capsules with a tasteless coat shell or sugar film. Sadly, in paediatric and geriatric treatment often only liquid forms of pharmaceuticals can be applied for instance due to swallowing problems. To overcome or minimize the bitterness of API several types of taste masking techniques are used [121] e.g., addition of sweeteners and artificial flavours. Bitterness, as well as taste masking components, can lead to hypersensitivity thus, their evaluation became an important quality-control parameter in the process of formulation of new medicines [122]. 

Bitterness intensity of peptides increases with the increase of their molecular weight and hydrophobicity [123]. However, according to our best knowledge, a theoretical prediction of the taste of new chemical compounds is not yet possible. Initially, panels of human volunteers were asked to describe the taste feelings. Human testing panels can be partially substituted by laboratory animals, particularly mice and rats. However, the ethical side of the utilization of human test panels and laboratory animals for the determination of taste of drugs on their early stage of development is questionable as it can be life-threatening in case of allergies. Moreover, results from human test panellists vary between individuals due to the high complicity of flavours and in case of small test groups provides only subjective information [123]. The perception of taste can differ depending on age, sex and the origin of a tester [123,124]. Expert paediatric panels are clearly unimaginable and although children are sometimes asked to participate in studies of commercially available drugs, which were already prescribed by physicians, their responses show great variability. A critical review of methodological limitations of protocols intended to assess taste-masking efficacy in view of the EU legislation was published recently [125].

To fulfil the need for an artificial tool for taste determination meeting ethical standards the electronic tongue was proposed. Studies on a correlation between mice and/or human test panels and an electronic tongue have been already reported [123] and led to creation of two commercial systems (Alpha MOS AstreeII, Toulouse, France and Intelligent Sensor Technology Inc., Atsugi-shi, Kanagawa, Japan, TS-5000Z) devoted to the analysis of API taste masking. A comparison between the commercially available and laboratory versions of electronic tongues for analysis of taste masking was presented in a review by Woertz [120]. Discussion on the influence of sweeteners, ion exchanging resins and complexing agents on bitterness in API also has been analysed [126]. 

New sensor types able to evaluate API taste masking strategies are constantly being created. A potentiometric sensor array was applied for discrimination between common API such as Ibuprofen and Rixithromycin and showed that microencapsulation exerted a similar effect on taste masking of both compounds [127]. Study on the bitter taste of sodium caseinate hydrolysates, which are a rich source of bioactive peptides and use of sweeteners as masking agents was proposed by Newman et al. [128]. An interesting study was recently presented by Pein et al. [129] in which groups of major specialists working in the area of electronic tongues, joined forces to compare commercially available systems with six sensor arrays developed in their laboratories. Nine formulations of different masking agents were evaluated under blind conditions, with data treated in a uniform way to provide an unbiased comparison. Results revealed similarities between arrays based on potentiometric sensors, with voltammetric system showing more distinct clustering pattern. Interestingly dummy samples containing caffeine citrate could be differentiated from API-free samples only by means of two systems, a potentiometric electronic tongue for static measurements and the voltammetric system. 

## 7. Other Applications

As we already mentioned, the application range of electronic tongue systems extends far beyond the analysis of food products and the evaluation of taste masking strategies. Numerous advantages such as speed and low cost of the analysis, as well as simplicity of the system and in many cases possibility of assembling of an electronic tongue from available sensors make it a promising tool for a variety of research areas. In this section, we would like to describe some of the other application trends of electronic tongue systems.

### 7.1. Biomedical Research

Although electronic tongue systems may be contributive to disciplines ranging from physical and analytical chemistry through nanotechnology, biology, pharmacokinetics to computational sciences their main application outside food industry is observed in biomedical research.

#### 7.1.1. Analysis of Biological Fluids 

Due to the specificity and technical plasticity of the electronic tongue, this system can be used as a diagnostic approach to identify and monitor early stages of pathological biological processes contributing to a broad spectrum of diseases. Target compounds can be detected regardless the type of biological fluids including urine, serum, blood or sweat. 

An electronic tongue enables the correct differentiation of urine samples from healthy and unhealthy patients suffering from renal dysfunction, the tool is particularly useful for identification of early stages of the disease. Several systems were already proposed for clinical analysis of urine, successfully predicting the quantity of ions (Na^+^, K^+^, NH_4_^+^, Ca^2+^, Mg^2+^, Cl^−^, SO_4_^2−^, PO_4_^3−^, H^+^/OH^−^: pH), urate and creatinine [130,131]. The system presented by Yaroshenko et al. [130] was applied to analysis of samples from 136 patients! both healthy and suffering from urolithiasis. Electronic tongue systems are particularly useful as a new technique for clinical monitoring of kidney diseases and urinary tumours [132,133]. 

Blood is one of the body fluids most widely used in diagnostics. The main advantage of using electronic tongue systems with this kind of complex samples is the elimination of interferents and thus contributing to an increase in the accuracy of the analysis. Nowadays, the main development in this field and at the same time a promising scientific direction is monitoring of haemodialysis processes in real-time [134]. In this area the most remarkable are systems composed of disposable implantable all-solid-state ion-selective electrodes for endoscopic studies, which were described by Tahirbegi et al. [135,136].

Monitoring of sweat is yet another potential application. In this case an electronic tongue could be based on tattoo sensors already described in the low-cost section. They can be positioned at different areas of the body for real-time monitoring of ions, glucose, lactic acid etc. during perspiration [85,86,87]. In this case electronic tongue technology could diminish the influence of the lag-time (up to 45 min in case of glucose), hydration of the organism, amount of perspiration etc. all being the source of uncertainty during sweat and interstitial fluid analysis [137]. This kind of a wearable tattoo-sensor could be a promising tool for monitoring performance of athletes or for detecting metabolic disorders. 

Disposable screen-printed sensors capable of quantifying chloride in sweat can be applied for the early detection of cystic fibrosis—a genetic disorder of endocrine glands affecting mostly the lungs, pancreas, liver, kidneys and intestine [138]. In case of sweat analysis an interesting direction is the possible fusion of electronic tongue systems with smart watches. Samsung released a product called Samsung Gear, which is equipped in slots that for now include physical sensors but are intended to be customizable and in principle will involve also biochemical sensors. As the programmers’ environment is available for free (http://developer.samsung.com/gear), algorithms for intelligent analysis of the data provided by such sensor array can be easily implemented. 

#### 7.1.2. In Vitro and In Vivo Studies

Miniaturized and mass producible pH and K^+^ sensor were applied for ischemia detection on stomach tissue. The sensor may be inserted directly into the stomach by an endoscopic device indicating a possibility of an in vivo measurement with the use of an electronic tongue systems. In this way, sensor arrays may be designed for evaluation of ischemic and reperfusion states of damaged tissue [135,136]. 

**Figure 6:**
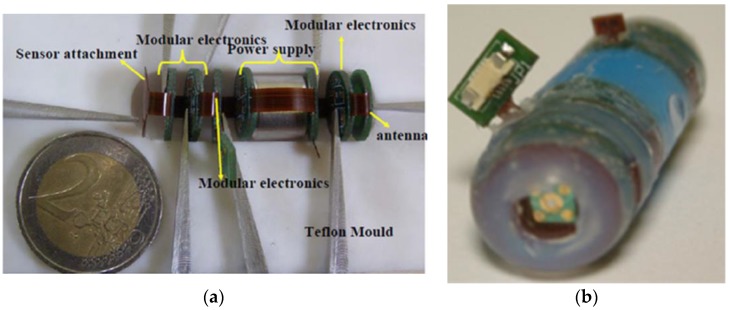
Sensor matrix in the form of a swallowable capsule. (**a**) prototype before packaging in the capsule, showing individual layers of the device; (**b**) final device packaged in polyether ether ketone; 12 mm × 28 mm (reprinted with permission from [139] ^©^Elsevier).

Swallowable capsules are becoming promising tools for the investigation of the gastrointestinal tract. Recently presented system of this type (Figure 6), communicates wirelessly, can perform multiple electrochemical assays and was already tested in 10 human subjects. Working on gut fluid, this miniaturized electronic tongue could aid diagnosis of complaints such as ulcerative colitis and Crohn’s disease [139]. 

**Figure 7:**
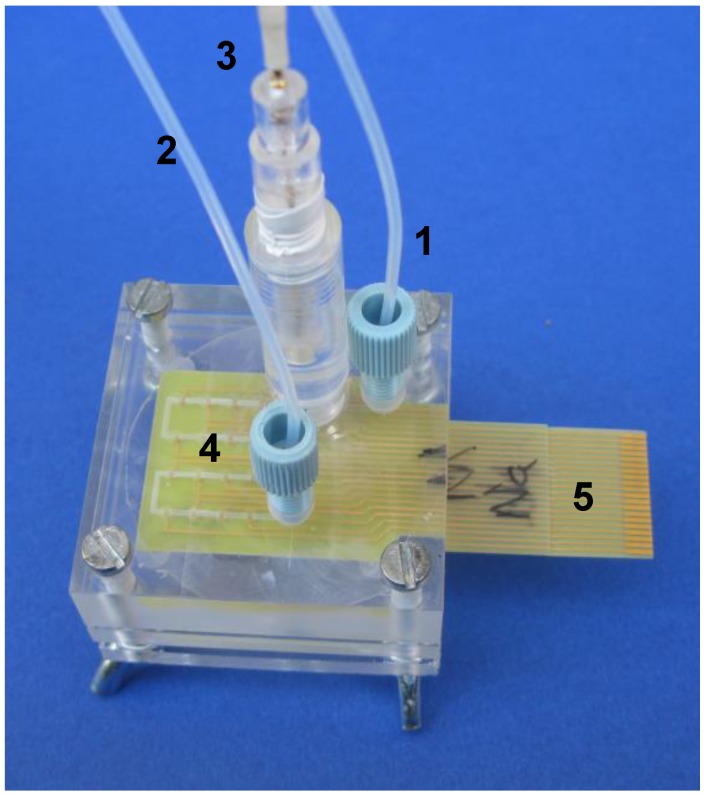
An electronic tongue system with integrated sensing matrix applied for an off-line analysis of cell cultures. 1. inlet, 2. outlet, 3. reference electrode, 4. integrated sensor matrix, 5. electrical connections (reprinted with permission from [140] ^©^Elsevier).

Due to the scarce availability of non-invasive chemical sensors, the electronic tongue may be an interesting alternative for monitoring vital signs of cells by detection of specific culture compounds in in vitro studies. An array of potentiometric sensors was applied for cytotoxicity assessment (Figure 7). As the analysis was based on used culture media discarded after daily passage of the cells, it can be considered a truly non-invasive method [140]. This kind of systems are a step forward towards automated drug screening using cell cultures which could replace animal testing. Thanks to their versatility sensor arrays have a potentially wide spectrum of analytical and medical applications, from a single cell to animal models. Nevertheless, for serious contributions to clinical screening, diagnosis or treatment to take place electronic tongues have to meet a series of stringent regulations applicable to medical devices. 

### 7.2. Safety

#### 7.2.1. National Safety 

Recently with the increased number of terrorist attacks, more attention is paid to explosive compounds. Development of new analytical techniques is particularly important to create more sensitive, faster and less expensive tools to determine explosives [141]. Specifically, screening of different unknown samples in search for a variety of dangerous compounds is highly demanded in areas of governmental protection and study of criminal cases.

Explosive substances are categorized in four major classes: nitrate esters, nitroamines, nitroaromatics and their peroxides [142,143]. Majority of currents analytical approaches are based on identification of structural carbon and nitrogen and are challenging in case of peroxides e.g., well known triacetone triperoxide. Peroxide explosives compounds are characterized by lack of chromophores and structural instability thus cannot be detected by other methods e.g., spectroscopy [144,145,146]. Due to these chemical features, peroxides are actually the main direction of terrorist explosive material development [147] indicating the necessity of elaboration of appropriate detection methods for this class of compounds. Electronic tongue systems are not only able to identify different nitrogen and nitroaromatic compounds but also, as recently demonstrated, peroxides [148,149,150,151,152,153,154].

In addition, the electronic tongue can be easily miniaturized and used in on-site tests [153,155] in this way being a promising tool for the detection and quantification of different explosive compounds. The next challenge is simultaneous analysis of all abovementioned classes of explosives using modified sensor matrixes [148]. 

#### 7.2.2. Environmental Safety

Rapid industrial growth produces many harmful and toxic chemicals, which are being released in the environment contributing to soil, air and water pollution. Some of the toxic chemical compounds are not biodegradable leading to accumulation in animal and plant tissues. Exposure to ozone, carbon monoxide, nitrogen dioxide, formaldehyde can among other affect respiratory, cardiovascular and nervous systems leading to asthma, asphyxiation, chemical anoxia, methemoglobinemia and different types of cancer (lung, nasopharynx, oropharynx) [21,156,157,158,159]. Early detection of toxic air and water pollutants in fast-growing cities can prevent harmful environmental consequences e.g., smog formation or its reduction creating also an interesting application for electronic nose systems [21,160].

The presence of nitrogen and phosphorus compounds above ppm levels increases the production of biomass in aquatic systems, thereby impairing the water quality and threatening the ecosystems balance [21]. Nitrite may also be the origin of certain types of cancer through the formation of nitrosamide and nitrosamine compounds [21,143,161]. Determination of the exact ratio of different forms of inorganic nitrogen species can be a useful indicator of the ecosystems condition. Therefore, the simultaneous determination of ammonium, nitrite and nitrate which was achieved by means of a potentiometric electronic tongue has a significant impact on environmental chemistry [21]. In another study pulse voltammetry performed on an array of metal electrodes was used to quantify ammonium nitrate in environmental water samples [143]. 

Detection of toxic substances, microorganisms and heavy metal ions in fresh water is another crucial aspect of water control. Presence of mentioned contaminants in tap or environmental water might be hazardous for both public health and animal life. In 2012 determination of nanomolar concentrations of organophosphorus insecticides (Chlorpyriphos-oxon, Chlorfenvinphos and Azinphos-methyl oxon) in river water samples was achieved by means of an array of biosensors [162]. Another system of this type was proposed recently [118] and was able to quantify organophosphate pesticides at nanomolar concentrations. Triclosan—an antibacterial agent, which although resulting in extremely harmful degradation products, is widely found in lakes and rivers was quantified by means of array system in pico to micromolar range [163]. 

Lately, an optical sensor array capable to identify diverse intermediate products of chemical industry, drugs and pesticides at the concentration levels below μm/L has been reported. Sensing layers based on porphyrins and pH indicators which interact with a wide range of analytes enabled a comprehensive analysis of tap water samples [164]. A potentiometric electronic tongue was applied for the detection of microcystin toxins (LOD 1 μg/L) released by cyanobacteria. Results obtained by means of the array system were with good agreement with standard chromatographic and colorimetric enzymatic tests [165]. Anodic stripping voltammetry performed on modified screen-printed electrodes allowed for the simultaneous determination of 4 heavy metal ions Cd(II), Pb(II), Tl(I) and Bi(III) in the presence of interferences (Zn(II), In(III). Thanks to appropriate signal processing it was possible to resolve overlapping peaks, which resulted in final correlation coefficients above 0.9 [166]. 

Besides the control of the freshwater parameters, quality of wastewaters also needs to be evaluated. In this context, a miniaturized bio-electronic tongue was applied towards the quality assessment of wastewater samples at different purification stages in a wastewater treatment plant. Amperometric measurements were performed in a flow-injection system and after signal processing samples were distinguished in terms of pollutant content. Additionally, commonly used organic pollutant indexes such as Microtox^®^, algae test, chemical oxygen demand, biochemical oxygen demand and total organic carbon were predicted [59]. The concentration of ammonia, nitrates as well as total soluble phosphates, chemical oxygen demand and conductivity could be also determined by means of a voltammetric electronic tongue [167].

## 8. Conclusions

The unique capabilities of electronic tongue systems, such as the ability to deal with complex and changing background and diminish the impact of interferents are the reasons of their paramount importance. Different sensing techniques, possible use of unconventional fabrication methods and numerous data treatment procedures indicate that electronic tongue systems can be tailored to various application areas. Moreover, precise and rapid analysis not requiring specially trained personnel make them a promising alternative for time-consuming and expensive analytical methods. Sensing arrays presented in this manuscript are developed to solve real-life problems ranging from humble analysis of foodstuffs’ quality, to far-fetched applications such as clinical analysis performed with swallowable capsules, endoscopic measurements in vivo or even identification of explosive compounds in relation to the current problem of terroristic threats. The coming years may disseminate this kind of systems even further, bringing them closer to consumers thanks to specific and advanced technology included in smart tools such as personalized smart watches or disposable low-cost sensor systems.
